# Occurrence of pendelluft during ventilator weaning with T piece correlated with increased mortality in difficult-to-wean patients

**DOI:** 10.1186/s40560-024-00737-z

**Published:** 2024-06-24

**Authors:** Wanglin Liu, Yi Chi, Yutong Zhao, Huaiwu He, Yun Long, Zhanqi Zhao

**Affiliations:** 1 State Key Laboratory of Complex Severe and Rare Disease, Department of Critical Care Medicine, Peking Union Medical College Hospital, Chinese Academy of Medical Sciences, 1 Shuaifuyuan, Dongcheng District, Beijing, China; 2 Department of Critical Care Medicine, The First Clinical Medical College of Shanxi Medical University, Taiyuan, China; 3 School of Biomedical Engineering, Guangzhou Medical University, No. 1 Xinzao Rd. Panyu District, Guangzhou, China; 4 Institute of Technical Medicine, Furtwangen University, VS-Schwenningen, Germany

**Keywords:** Difficult-to-wean, Pendelluft, Electrical impedance tomography, Mortality

## Abstract

**Background:**

Difficult-to-wean patients, typically identified as those failing the initial spontaneous breathing trial (SBT), face elevated mortality rates. Pendelluft, frequently observed in patients experiencing SBT failure, can be conveniently detected through bedside monitoring with electrical impedance tomography (EIT). This study aimed to explore the impact of pendelluft during SBT on difficult-to-wean patients.

**Methods:**

This retrospective observational study included difficult-to-wean patients undergoing spontaneous T piece breathing, during which EIT data were collected. Pendelluft occurrence was defined when its amplitude exceeded 2.5% of global tidal impedance variation. Physiological parameters during SBT were retrospectively retrieved from the EIT Examination Report Form. Other clinical data including mechanical ventilation duration, length of ICU stay, length of hospital stay, and 28-day mortality were retrieved from patient records in the hospital information system for each subject.

**Results:**

Pendelluft was observed in 72 (70.4%) of the 108 included patients, with 16 (14.8%) experiencing mortality by day 28. The pendelluft group exhibited significantly higher mortality (19.7% vs. 3.1%, *p* = 0.035), longer median mechanical ventilation duration [9 (5–15) vs. 7 (5–11) days, *p* = 0.041] and shorter ventilator-free days at day 28 [18 (4–22) vs. 20 (16–23) days, *p* = 0.043]. The presence of pendellfut was independently associated with increased mortality at day 28 (OR = 10.50, 95% confidence interval   1.21–90.99, *p* = 0.033).

**Conclusions:**

Pendelluft occurred in 70.4% of difficult-to-wean patients undergoing T piece spontaneous breathing. Pendelluft was associated with worse clinical outcomes, including prolonged mechanical ventilation and increased mortality in this population. Our findings underscore the significance of monitoring pendelluft using EIT during SBT for difficult-to-wean patients.

**Supplementary Information:**

The online version contains supplementary material available at 10.1186/s40560-024-00737-z.

## Introduction

Patients with difficulty in weaning from invasive mechanical ventilation were found to have worse clinical outcomes, including increased mortality [1, 2]. Spontaneous breathing trials (SBT) are essential for assessing a patient’s readiness to withdraw from the ventilator by allowing them to breathe with minimal or no assistance [3]. SBT can be conducted using either low-level pressure support ventilation or a T piece [4, 5]. Patients who had difficulty in weaning are typically identified as those who fail the initial SBT and require two or more SBTs to achieve successful weaning, in accordance with the Sixth International Consensus Conference on Intensive Care Medicine’s definition [5]. The pathophysiology of weaning failure is intricate [6]. Recent studies have illuminated the significance of pendelluft, which can be assessed through electrical impedance tomography (EIT) during the SBT process. Pendelluft is frequently observed in patients who fail an SBT or can even serve as a predictor of weaning failure [7, 8]. EIT is a non-invasive and radiation-free imaging technique that enables the visualization of regional ventilation [9–11]. It has found extensive use in investigating the causes of hypoxemia in critically ill patients [12, 13].

Pendelluft, defined as the redistribution of intrapulmonary gas without a significant change in tidal volume, plays a crucial role in patient self-inflicted lung injury [15]. It has even been proposed as a surrogate marker for vigorous inspiratory effort [16]. The presence of pendelluft can be conveniently detected through bedside EIT monitoring [7, 8, 14, 15]. A recent study demonstrated that pendelluft occurred during high-flow nasal oxygen therapy could be alleviated by continuous positive airway pressure and further improved with noninvasive ventilation, along with reduced inspiratory effort [17]. The phenomenon of pendelluft has predominantly been observed in mechanically ventilated patients or those undergoing SBT with low-level pressure support ventilation settings [7, 8, 14–16]. These studies revealed the link between pendelluft and reduced ventilatory efficiency as well as weaning failure. Our previous research demonstrated that pendelluft was associated with prolonged mechanical ventilation duration in ICU patients with mild-to-severe acute respiratory failure [14]. However, no prior investigations had explored the impact of pendelluft during the spontaneous T piece breathing process on difficult-to-wean patients. Furthermore, existing literature lacked evidence regarding the association between pendelluft and mortality.

Hence, this retrospective observational study aimed to investigate the influence of pendelluft during SBT using a T-piece on difficult-to-wean patients. Our study hypothesis was that the presence of pendelluft would correlate with increased mortality rate in this specific patient population.

## Methods

### Patients

From July 2021 to June 2023, consecutive intensive care unit (ICU) patients who were difficult to wean from invasive mechanical ventilation, received EIT examinations during the SBT process were eligible for study inclusion. Difficult-to-wean patients were those defined as individuals who did not succeed in their initial SBT with a T-piece and subsequently needed to undergo additional SBTs, or took up to 24 h from the commencement of the first SBT to achieve successful weaning. Reintubation within 48 h would be considered as failure and categorized as difficult-to-wean as well. Prior to SBT, which was conducted using a T piece in our department, PEEP and pressure support levels were adjusted to 5–6 cmH_2_O and 8–10 cmH_2_O, respectively. If the patients were stable, SBT was followed. In our department, EIT examination was performed by an independent EIT team based on the clinical requirement. The EIT assessment was triggered by hypoxemia as a routine practice in our department. Patients not exhibiting hypoxemia typically did not undergo EIT assessment. In this study, all patients underwent EIT assessment due to varying degrees of hypoxemia. Specifically, those who were difficult to wean from invasive mechanical ventilation and received EIT examinations during the SBT process using a T-piece were selected for analysis. In cases patients underwent multiple SBTs, only the first SBT during which EIT measurement was conducted would be included for analysis. The standard SBT duration in our department was typically 2 h. However, if a patient remained stable during the initial 2 h but did not meet extubation criteria, the trial could be extended to 4–8 h based on the patient’s condition. If a patient experienced breathing difficulties during the SBT, the ventilator was promptly reconnected. Any interruption of the SBT for reasons other than cardiac-pulmonary issues (e.g., increased sedation due to patient delirium or agitation) was considered a single trial instead of two. Upon meeting extubation criteria assessed by the attending physician, the endotracheal tube would then be removed after 2 h of SBT. Extubation criteria included SpO_2_ > 95%, respiratory rate < 30 breaths/min, hemodynamically stable, presence of cough reflex, presence of cuff leak, and the ability to protect the airway. Reintubation was performed if the patients presented difficulties in breathing and could not be allievated by non-invasive positive pressure ventilation. The exclusion criteria were: age < 18 years, pregnancy, body mass index over 50 kg/m^2^, ribcage malformation. This retrospective study was approved by the Institutional Research and Ethics Committee of Peking Union Medical College Hospital (K5289). Informed consent was not applicable for the retrospective study.

### Clinical data collection

Acute physiology and chronic health evaluation (APACHE) II score were collected for the first 24 h of ICU admission. The arterial blood gas analysis data were retrieved from the hospital information system on the same day as the SBT. Arterial blood gas analysis was performed prior to the SBT in the majority of our patients. For those patients who did not undergo arterial blood gas analysis before the SBT, data from arterial blood gas analyses after the SBT were retrieved from patient records in the hospital information system. The patients’ physiological variables, including heart rate (HR), mean arterial blood pressure (MAP), respiratory rate (RR), pulse oxyhemoglobin saturation (SpO_2_) and the fraction of inspired oxygen (FiO_2_) were obtained at the moment of EIT assessment during SBT by the independent EIT team, who filled in an EIT Assessment Report Form with these physiological variables for each assessment based on local regulation. Moreover, partial pressure of arterial oxygen (PaO_2_)/FiO_2_ ratio, SpO_2_/FiO_2_ ratio and ROX index (defined as the ratio of SpO_2_/FiO_2_ to RR) [18] were calculated. The mechanical ventilation duration preceding the EIT assessment, total mechanical ventilation duration, ventilator-free days (VFDs) at day 28 [19], length of ICU stay, length of hospital stay, 28-day mortality, as well as the dates of intubation and extubation or tracheotomy, were retrieved from patient records in the hospital information system. VFDs were calculated as the number of days without invasive ventilation to day 28. Non-survivors were considered to have a VFDs value of 0. The causes of death for the non-survivors were also retrieved and recorded from patient records in the hospital information system.

### EIT assessment

The EIT assessment was triggered by hypoxemia as a routine practice in our department. Patients not exhibiting hypoxemia typically did not undergo EIT assessment. In this study, all patients underwent EIT assessment due to varying degrees of hypoxemia. EIT assessments with PulmoVista 500 (Dräger Medical, Lübeck, Germany) were conducted approximately 30 to 60 min after the initiation of T piece spontaneous breathing. An EIT belt with 16 electrodes was placed around the patient’s thorax at the 4–5th intercostal space level. When patients underwent multiple EIT examinations during various SBTs, only the initial EIT data during SBT were utilized for analysis. The EIT data were digitally filtered using a low-pass filter with a cutoff frequency of 0.67 Hz (or 40/min), to eliminate cardiac-related impedance changes. The data were analyzed offline using customized software programmed with MATLAB R2015 (The MathWorks Inc., Natick, MA, USA). To reduce the high variability among breaths during SBT, 2-min continuous EIT data were recorded, and the resultant average data was utilized for analysis. The EIT-based pendelluft amplitude was defined as the impedance difference between the sum of all regional tidal impedance variation (TIV) and the global TIV (show in Figure S1) based on a previous study [20]. Due to the potential noise influence on this pixel-based algorithm, the occurrence of pendelluft was considered when its amplitude exceeded 2.5% of global TIV (which was the 95th percentile from 30 healthy volunteers based on our previous study) [14].

### Statistical analysis

To explore the difference in mortality rates between patients with pendelluft and those without pendelluft, a sample size calculation was performed. Given the absence of prior studies that specifically reported mortality among patients experiencing both pendelluft and difficulty in weaning, we referred to the varying mortality rates within the difficult-to-wean population, which ranged from 19.0% to 36.8% [1]. Wang et al. previously reported a 28-day mortality of 70% for patients who failed SBT, compared to 4% for those who succeeded [8]. Similarly, our previous study indicated a mortality rate of 16% for patients with acute respiratory failure and pendelluft, contrasting with 9% for those without pendelluft [14]. Guided by these insights, we conservatively assumed a mortality rate of 40% for the pendelluft group and 15% for the no pendelluft group in our difficult-to-wean population. With a significance level (alpha) set at 0.05 and a desired power (1-beta) of 0.80, a sample size calculation was conducted using a Chi-squared test. The analysis determined that a minimum sample size of 105 patients would be required to detect a significant difference in mortality rates between the two groups. This sample size ensures that the study has adequate statistical power to draw meaningful conclusions regarding the impact of pendelluft on patient outcomes.

Data were presented as median and interquartile range (25th–75th percentiles) for non-normal distribution parameters and as numbers and percentages for categorical variables. Categorical variables were compared using either the Chi-square test or Fisher’s precision probability test, as appropriate. Continuous variables between groups were compared using the Mann–Whitney *U* test for nonparametrically distributed data and the Student *t* test for parametrically distributed data. Multivariable logistic regression analysis was conducted to examine the relationship between the occurrence of pendelluft and 28-day mortality while adjusting for variables such as sex, age, APACHE II score, SpO_2_/FiO_2_ ratio, PaO_2_/FiO_2_ ratio, and the duration of mechanical ventilation prior to the EIT assessment. Given the limited number of non-survivors (*n* = 16), the selection of covariates was constrained. Consequently, logistic regression models were employed with various combinations of variables to explore the potential cofounding factors. *p* value < 0.05 was defined as statistically significant. All the statistical analyses were performed using the SPSS 22.0 software package (SPSS, Chicago, IL) and R version 4.0.3.

## Results

### Patients clinical characteristics

**Fig. 1:**
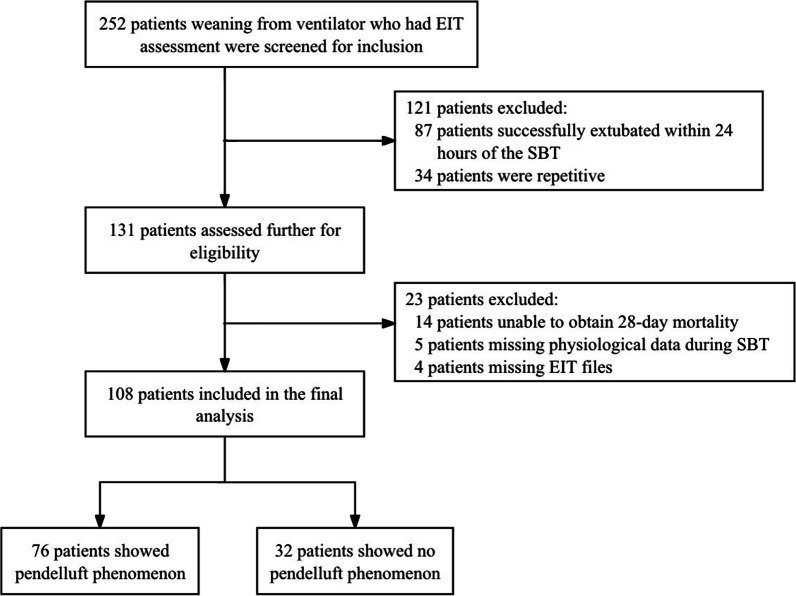
Study flow chart

A total of 108 patients who met the inclusion criteria were analyzed in our study (Fig. 1). The clinical characteristics of all the subjects are shown in Table 1. Of the 108 patients, 92 (85.2%) patients survived while 16 (14.8%) patients died. Seventy-eight (72.2%) patients were male. The median age of all the subjects was 64 (54–71) years. Seventy-five (69.4%) patients were admitted to ICU post major surgery (including cardiac surgery, major abdominal surgery, tri-incisional esophagectomy and major thoracoscopy surgery). The remaining 33 (30.6%) patients were internal medical patients who were admitted to ICU due to respiratory failure caused by lung infection or septic shock. The underlying causes of hypoxemia for the patients are demonstrated in Table S1. Median APACHE II score were 17 (14–20) on ICU admission and 14 (12–18) at EIT assessment. Median PaO_2_/FiO_2_ ratio was 307 (243–379) mmHg.

**Table 1:** Clinical characteristics of the subjects and comparisons between patients with and without pendelluft phenomenon

Characteristics	All subjects (*n* = 108)	Pendelluft group (*n* = 76)	No pendelluft group (*n* = 32)	*p*
Age, years	64 (54–71)	64 (56–71)	63 (50–70)	0.440
Sex, male, *n* (%)	78 (72.2%)	52 (68.4%)	26 (81.3%)	0.174
BMI,	25 (22–27)	24 (22–27)	25 (23–26)	0.399
Causes of ICU admission				
Postcardiac surgery	49 (45.3%)	33 (43.4%)	16 (50.0%)	0.411
Post noncardiac surgery	26 (24.1%)	21 (27.6%)	5 (15.6%)	
Respiratory failure	33 (30.6%)	22 (28.9%)	11 (34.4%)	
RR*, Bpm	20 (16–24)	20 (16–25)	21 (18–24)	0.853
SpO_2_*, %	99 (97–100)	99 (97–100)	100 (97–100)	0.817
FiO_2_*, %	31 (30–35)	31 (30–35)	31 (30–35)	0.757
PaO_2_^a^, mmHg	97 (82–118)	98 (84–118)	96 (74–115)	0.526
PaCO_2_^a^, mmHg	39 (36–42)	39 (35–41)	40 (38–43)	0.057
PaO_2_/FiO_2_^a^, mmHg	307 (243–379)	307 (244–381)	307 (217–364)	0.560
SpO_2_/FiO_2_*	3.20 (2.86–3.33)	3.20 (2.86–3.33)	3.21 (2.80–3.31)	0.902
ROX*	15.10 (12.50–19.03)	14.72 (12.31–20.16)	15.26 (12.8–17.98)	0.981
Pendelluft amplitude, %	4.20 (1.92–11.63)	7.35 (3.75–16.58)	1.35 (0.67–1.90)	< 0.001
HR*, bpm	92 (84–101)	93 (82–100)	92 (88–103)	0.185
MAP*, mmHg	85 (78–96)	85 (78–94)	89 (78–98)	0.648
APACHE II on admission day	17 (14–20)	17 (14–21)	16 (14–20)	0.358
APACHE II at EIT assessment	14 (12–18)	14 (12–17)	14 (11–19)	0.630
MV days preceding EIT assessment	5 (2–9)	6 (2–9)	4 (2–6)	0.124
MV days in total	8 (5–14)	9 (5–15)	7 (5–11)	0.041
VFDs at day 28	18 (10–22)	18 (4–22)	20 (16–23)	0.043
ICU stay, days	13 (7–20)	13 (8–21)	12 (7–18)	0.063
Hospital stay, days	27 (18–38)	28 (18–43)	24 (18–35)	0.059
Death at day 28, *n* (%)	16 (14.8%)	15 (19.7%)	1 (3.1%)	0.035

Data were presented with Median (Interquartile Range). *BMI* Body Mass Index, *MV* mechanical ventilation, *VFDs* ventilator-free days, *HR* hear rate, *Bpm* breaths per minute, *bpm* beats per minute, *RR* respiratory rate, *SpO*_*2*_ pulse oxyhemoglobin saturation, *FiO*_*2*_ fraction of inspired oxygen, *PaO*_*2*_ partial pressure of arterial oxygen, *PaCO*_*2*_ partial pressure of arterial carbon dioxide, *ROX* SpO_2_/FiO_2_/RR, *MAP* mean arterial blood pressure, *APACHE II* acute physiology and chronic health evaluation II

*These parameters were measured during the SBT process

^a^These parameters were not measured during the SBT process but on the same day as the SBT

Twenty-four patients (22.2%) underwent EIT assessments during their initial SBT, 37 patients (34.3%) during their second SBT, 15 patients (13.9%) during their third SBT, 9 patients (8.3%) during their fourth SBT, and 5 patients (4.6%) during their fifth SBT. The remaining 16.7% of patients underwent EIT assessment during their 6th to 15th SBT sessions. The median duration of SBT was 8 (4.25–8) h in our study.

The causes of death of the non-survivors were as follows: 8 patients died of severe pneumonia and septic shock, 2 patients died of mediastinal infection following cardiac surgery, 2 patients died of abdominal infection following major abdominal surgery, 2 patients died of cardiac shock following cardiac surgery, and 2 patients died of primary cervical and mediastinal abscesses.

### The outcome of the SBT

Out of the total cohort, 64 patients (59.3%) underwent successful extubation, 39 patients (36.1%) were tracheotomized (11 of whom did not survive), 1 patient was reintubated and died later, and the remaining 4 patients died before undergoing either tracheotomy or extubation. Due to the retrospective design of the study, 19 of the 39 tracheotomized patients were already in a tracheostomy state when the EIT assessment during SBT was conducted. Among the total cohort, pendelluft was observed in 40 out of the 64 patients who were successfully extubated, whereas it was present in 36 out of the 44 patients who were not (62.5% vs. 81.8%, *p* = 0.031). Among the 92 patients who survived, a Chi-square test revealed no significant difference in the occurrence of pendelluft between those who were successfully extubated and those who underwent tracheotomy (62.5% vs. 75.0%, *p* = 0.354).

### The comparison between patients with pendelluft and without pendelluft

Pendelluft occurred in 76 (70.4%) patients and was absent in 32 (29.6%) patients. In addition, Fisher’s precision probability test showed significant higher mortality in patients with pendelluft (the pendelluft group) than in those without pendelluft (the no pendelluft group) (19.7% vs. 3.1%, *p* = 0.035) (Table 1). Compared to patients without pendelluft, patients with pendelluft exhibited longer total mechanical ventilation duration [9 (5–15) vs. 7 (5–11) days, *p* = 0.041] and shorter VFDs to day-28 [18 (4–22) vs. 20 (16–23) days, *p* = 0.043] (Table 1). The length of ICU stay [13 (8–21) vs. 12 (7–18) days, *p* = 0.063] and hospital stay of [28 (18–43) vs. 24 (18–35) days, *p* = 0.059] tended to be longer in the pendellfut group) (Table 1). Nevertheless, no significant differences were observed between the two groups regarding RR, SpO_2_/FiO_2_ ratio, ROX index, HR, MAP, disease severity as assessed by APACHE II on ICU admission day and at EIT assessment, as well as the PaO_2_/FiO_2_ ratio at the same day as the EIT assessment.

### The association between the occurrence of pendelluft and the risk of mortality

To explore the potential cofounding factors, different logistic models were performed using different combinations of variables (Table 2). Multivariate logistic regression analysis showed that the occurrence of pendellfut was independently associated with increased mortality at day 28 (OR = 10.50, 95% CI   1.21–90.99, *p* = 0.033), after adjusting for sex, age, SpO_2_/FiO_2_ ratio at EIT assessment and APACHE II score on ICU admission day (Table 2). After adjusting for sex, age, PaO_2_/FiO_2_ ratio, APACHE II score and days of mechanical ventilation before EIT assessment, the occurrence of pendelluft was still independently associated with increased mortality at day 28 (OR = 8.90, 95% CI   1.08–73.32, *p* = 0.042) (Table 2).

**Table 2:** Multivariate logistic regression analysis for mortality at day 28

Variables	B	S.E.	Wald	*p*	OR	95% Confidence Interval	
						Lower limit	Upper limit
Model 1							
Pendelluft	2.352	1.102	4.556	0.033	10.502	1.211	90.99
Sex	-1.412	0.837	2.848	0.091	0.244	0.047	1.256
Age	− 0.003	0.021	0.014	0.905	0.997	0.957	1.04
SpO_2_/FiO_2_	− 0.686	0.75	0.835	0.361	0.504	0.116	2.193
APACHE II*	0.048	0.04	1.422	0.233	1.049	0.969	1.136
Model 2							
Pendelluft	2.186	1.076	4.13	0.042	8.902	1.081	73.328
Sex	− 1.259	0.81	2.416	0.12	0.284	0.058	1.389
PaO_2_/FiO_2_	− 0.003	0.004	0.581	0.446	0.997	0.99	1.004
MV days^a^	0.013	0.016	0.657	0.418	1.013	0.981	1.046
APACHE II*	0.048	0.039	1.488	0.223	1.049	0.971	1.132
Model 3							
Pendelluft	2.17	1.07	4.113	0.043	8.756	1.076	71.273
Sex	− 1.284	0.805	2.544	0.111	0.277	0.057	1.342
PaO_2_/FiO_2_	− 0.003	0.004	0.503	0.478	0.997	0.99	1.004
MV days^a^	0.012	0.016	0.557	0.455	1.012	0.98	1.045
APACHE II^b^	0.004	0.051	0.005	0.942	1.004	0.909	1.108
Model 4							
Pendelluft	2.171	1.064	4.162	0.041	8.768	1.089	70.59
Sex	− 1.297	0.815	2.535	0.111	0.273	0.055	1.349
Age	0.005	0.021	0.062	0.803	1.005	0.965	1.047
Model 5							
Pendelluft	2.208	1.067	4.281	0.039	9.096	1.124	73.646
Sex	− 1.286	0.813	2.503	0.114	0.276	0.056	1.359
Age	0.006	0.021	0.067	0.795	1.006	0.965	1.048
PaO_2_/FiO_2_	− 0.002	0.003	0.31	0.578	0.998	0.992	1.005
Model 6							
Pendelluft	2.166	1.07	4.097	0.043	8.724	1.071	71.053
Sex	− 1.274	0.816	2.438	0.118	0.28	0.057	1.384
Age	0.002	0.022	0.008	0.93	1.002	0.96	1.046
PaO_2_/FiO_2_	− 0.002	0.004	0.491	0.483	0.998	0.991	1.004
MV days^a^	0.012	0.017	0.482	0.488	1.012	0.979	1.046

*APACHE II score on ICU admission day. ^a^Mechanical ventilation duration preceding the EIT assessment. ^b^APACHE II score on the day of EIT assessment. *SpO*_*2*_*/FiO*_*2*_ pulse oxyhemoglobin saturation/fraction of inspired oxygen ratio, *PaO*_*2*_*/FiO*_*2*_ partial pressure of arterial oxygen/fraction of inspired oxygen ratio, *MV* mechanical ventilation, *S.E.* standard error, *OR* odds ratio

## Discussion

Our study found that the occurrence of pendelluft during T piece spontaneous breathing happened in 70.4% ICU patients who were difficult to wean. The patients with pendelluft had significantly longer mechanical ventilation duration, shorter VFDs to day 28 and higher 28-day mortality compared with patients without pendelluft. Patients who were successfully extubated exhibited a significantly lower occurrence of pendelluft compared to those who were not. Our findings further revealed that the occurrence of pendelluft was independently associated with an increased risk of 28-day mortality among these patients.

Our previous study revealed a pendelluft prevalence of 31% in a population with acute respiratory failure under mechanical ventilation [14]. However, it’s important to note that EIT assessments were not conducted during SBT in that study. In our current study of difficult-to-wean population, we observed a notable increase in the prevalence of pendelluft, rising to 70.4% during SBT. The occurrence of the pendelluft may be attributed to factors such as alveolar heterogeneity or excessive spontaneous breathing effort, leading to gas movement within the lung [7, 8, 21, 22]. Throughout the weaning process, patients’ spontaneous breathing effort progressively intensifies, reaching its zenith when they are disconnected from the ventilator. During spontaneous breathing with a T piece, the local negative pleural pressure generated by diaphragmatic muscle contraction can result in inflation of the dependent lung region and simultaneous deflation of the nondependent lung region [23]. This dynamic could account for the significantly elevated prevalence of pendelluft, as observed in our study during SBT with a T piece. The more than twofold increase in pendelluft prevalence during SBT with a T piece in difficult-to-wean patients may also indicate a heightened risk of lung injury in this patient population. It is important to note that, despite the substantial prevalence of pendelluft, the patients included in our study were clinically deemed ready for weaning based on other respiratory parameters and their overall condition, as assessed by the attending intensivist at the time of EIT assessment. From this perspective, monitoring lung ventilation and the pendelluft using EIT during spontaneous T piece breathing could provide valuable additional insights into the actual respiratory function of these patients during the weaning process. EIT may serve as a non-invasive tool to help intensivists and respiratory therapists make more informed decisions regarding the timing of weaning, potentially reducing the risk of complications associated with premature weaning or prolonged mechanical ventilation. Further research and clinical validation are warranted to explore the full clinical utility of EIT in the weaning process.

Our study demonstrated for the first time that the presence of pendelluft independently correlated with increased mortality among difficult-to-wean patients. While Wang et al. previously showed that pendelluft during pressure support ventilation SBT could predict weaning failure [8], their study did not report mortality outcomes. As a retrospective study, we did not observe a significant difference in the occurrence of pendelluft between surviving patients who underwent successful extubation and those who underwent tracheotomy. However, we did detect a significant higher prevalence of pendelluft in non-survivors compared to survivors (93.8% vs. 66.3%). Our previous study found a correlation between the presence of pendelluft and prolonged mechanical ventilation but did not observe a difference in mortality among ICU patients with PaO_2_/FiO_2_ ratio below 200 mmHg [14]. The unique focus on difficult-to-wean patients in our current study may account for this new finding. In consistence with our previous findings, the pendellfut group in the difficult-to-wean patient population exhibited significantly longer mechanical ventilation duration and shorter VFDs to day 28 [14]. In addition, the length of ICU and hospital stay tended to be longer in the pendellfut group, providing further evidence of the observed increase in mortality within this subgroup. Though larger-scale, prospective studies are essential for validating our results, the clinical significance of monitoring pendelluft using EIT is underscored by its impact on mortality. Our findings call for future randomized controlled trials aimed at exploring methods to mitigate pendelluft in clinical practice and evaluating the associated benefits.

Pendelluft has been reported to disappear following the administration of muscle paralysis in mechanically ventilated patients with acute respiratory disease syndrome, as indicated in previous studies [22–24]. In the case of patients undergoing SBT using a T piece, application of neuromuscular blockers to attenuate diaphragm muscle contractions was infrequent and required close monitoring in clinical practice. However, in certain instances, low dosages of sedatives and analgesics were employed to mitigate excessive breathing effort, alleviate anxiety, and maintain an appropriate level of wakefulness, particularly in cases involving delirious patients [25–27]. Some patients included in our study received low-dose continuous fentanyl infusion (15–30 μg/h) during the spontaneous T piece breathing process. Unfortunately, given the retrospective nature of this study, we were unable to obtain the relevant parameters indicative of breathing effort or diaphragm muscle function. However, it is noteworthy that a considerable number of patients (62.5%) who were successfully extubated in our difficult-to-wean population exhibited pendelluft during SBT. A recent study demonstrated that pendelluft occurred during high-flow nasal oxygen therapy could be alleviated by continuous positive airway pressure and further improved with noninvasive ventilation, along with reduced inspiratory effort [17]. In our department, the use of high-flow nasal oxygen as a post-extubation strategy is quite common due to its enhancement in patient comfort. Noninvasive ventilation and continuous positive airway pressure could also be considered. Future investigations may be warranted to thoroughly explore the benefits of these noninvasive post-extubation strategies in the context of pendelluft and its impact on patient prognosis.

We did not find any significant differences in RR, SpO_2_/FiO_2_ ratio and ROX index during the SBT between patients with and without pendellfut. Similarly, the PaO_2_/FiO_2_ ratio measured not during SBT but on the same day as the SBT showed no statistically significant differences between the two groups. PaCO_2_ seemed to be slightly decreased in the pendelluft group without reaching statistical significance (39 vs. 40 mmHg, *p* = 0.057). Previous studies have reported the association between pendelluft and increased EtCO_2_ and RR [7, 14]. However, it’s important to consider that arterial blood gas analysis was not conducted simultaneously but on the same day as the SBT in our study, making it less reflective of real-time oxygenation and ventilatory efficiency. Therefore, the lack of significant difference in PaO_2_/FiO_2_ ratio and PaCO_2_ between the two groups can be understood. In contrast, the physiological parameters, including RR, SpO_2_/FiO_2_ ratio and ROX index were recorded at the time of EIT assessment during the spontaneous T piece breathing process. Consequently, the absence of significant difference in these parameters between patients with and without pendelluft in our study suggested that these traditional global respiratory indicators may not be very sensitive in reflecting lung inhomogeneities during SBT. Regional pendelluft assessment reflects the heterogeneity in lung mechanics and regional intrapleural pressure imbalances, which may offer valuable insights for assessing respiratory conditions.

Our study has several limitations that should be considered. The retrospective design was a major limitation of the study, which limits the ability to establish a causal relationship between pendelluft and mortality. It is crucial to recognize that the association between pendelluft and mortality may be influenced by factors linked to the underlying reasons for difficult weaning, which are beyond the study’s scope. In addition, investigating the correlation between pendelluft and ventilatory drive would be insightful. However, parameters such as airway occlusion pressure or maximum inspiratory pressure are not regularly measured, thus precluding this analysis. Second, the times of EIT examination during SBTs exhibited heterogeneity due to the retrospective nature of this study. Specifically, 83.3% of patients underwent EIT assessments before their 6th SBT. To reduce the potential impact of this variability, only the initial EIT data during SBT was included for analysis. Further study is required to explore the variation of pendelluft in various SBT stages. Third, the causes of death could be multifactorial and extend beyond respiratory failure. The absence of control arm and longitudinal data may not fully capture the comprehensive clinical effects of pendelluft and how it evolves over time. Regrettably, we were unable to obtain EIT data before or after the SBT due to the nature of retrospective analysis, as not every patient included in our study underwent EIT assessment prior to or following the SBT. However, it would be valuable to include EIT data before the SBT to see if there is a difference in the occurrence of pendelluft during SBT and ventilation in future studies. Fourth, the selection of parameters for inclusion in the multivariate binary logistic regression model was mainly based on two categories: demographic characteristics (i.e., age, sex) and clinical characteristics (i.e., pendelluft, SpO_2_/FiO_2_ or PaO_2_/FiO_2_, mechanical ventilation duration and APACHE II). Because the cases of non-survivors (*n* = 16) were low, the number of selected variables was limited to those we thought to be important and clinically relevant. Fifth, it may be argued that using pendelluft as a continuous variable might provide more precise results than treating it as a binary outcome. When pendelluft is lower than a certain threshold, it probably represents only noise and not real pendelluft. Therefore, it would introduce error to the regression model if pendelluft was used as a continuous variable. Besides, a much larger sample size is required to effectively utilize pendelluft amplitude as a continuous variable in the multivariate logistic regression analysis. Recently, Wang et al. reported a 3% cut-off to predict the weaning outcome [8]. In our study, we found a 2.5% cutoff value to define the occurrence of pendelluft related to mortality. A search for an optimal threshold for various purposes should be conducted in further studies. In addition, the sample size in our study was relatively small, which could potentially limit the statistical power and increase the risk of type II errors. The broad inclusion criteria and heterogeneity of the included patients may have introduced variability into the data, further affecting the statistical power of the study. Lastly, the restriction of including difficult-to-wean patients, while allowing us to focus on a specific subgroup, may limit the generalizability of our findings to a broader population and may cause bias to the result. However, patients who had no difficulty in weaning were usually successfully extubated after the first SBT and received no EIT measurement. Future prospective studies should consider enrolling this patient population to reduce bias. However, the focus on the difficult-to-wean population in the current study enables us to investigate the association between pendelluft and mortality in the group where this relationship was most likely to be observed.

## Conclusions

The occurrence of pendelluft was observed in 70.4% of difficult-to-wean patients undergoing T piece spontaneous breathing. Importantly, pendelluft was independently associated with adverse clinical outcomes, including increased mortality in this population. These findings underscore the significance of monitoring pendelluft using EIT during SBT for difficult-to-wean patients.

### Supplementary Information

Supplementary Material 1.

Supplementary Material 2.

## Data Availability

The datasets analyzed during the current study are available from the corresponding author on reasonable request.

## Abbreviations

SBT: Spontaneous breathing trial
APACHE: Acute Physiology and Chronic Health Evaluation
EIT: Electrical impedance tomography
ICU: Intensive care unit
HR: Heart rate
MAP: Mean arterial blood pressure
RR: Respiratory rate
SpO_2_: Pulse oxyhemoglobin saturation
FiO_2_: Fraction of inspired oxygen
PaO_2_: Partial pressure of arterial oxygen
VFDs: Ventilator-free days
TIV: Tidal impedance variation
CI: Confidence interval
OR: Odds ratio
