# Supplementary material for: Occurrence of pendelluft during ventilator weaning with T piece correlated with increased mortality in difficult-to-wean patients
Source: J Intensive Care. 2024 Jun 24;12:23. doi: 10.1186/s40560-024-00737-z (PMC11194869; doi:10.1186/s40560-024-00737-z)
Supplement: Supplementary file 1 — Supplementary Material 1. [file 40560_2024_737_MOESM1_ESM.docx]

Table S1. The causes of hypoxemia for patients.

| Cause s of ICU admission | Pendelluft group  (n = 76) | No pendelluft group  (n = 32) |
| --- | --- | --- |
| Postcardiac surgery  (n = 49) | (n = 33)  26= pulmonary atelectasis / consolidation  5= postoperative pneumonia  2= pneumothorax | (n = 16)  14= pulmonary atelectasis / consolidation  2= postoperative pneumonia |
| Post noncardiac surgery  (n = 26) | (n =21)  7= post abdominal surgery/ abdominal infection / acute respiratory distress syndrome  6= post abdominal surgery / pulmonary atelectasis/ consolidation  5= post thoracic surgery / pulmonary atelectasis/ consolidation  1= post lower limb vascular interventional surgery /severe postoperative pneumonia  2= post intracranial surgery / severe postoperative pneumonia | (n = 5)  1= post thoracic surgery / pulmonary atelectasis / consolidation  1= post abdominal surgery /abdominal infection /acute respiratory distress syndrome  1= post interventional surgery for aortic dissection /acute respiratory distress syndrome  1= post abdominal surgery /abdominal infection /severe postoperative pneumonia  1= pulmonary embolism |
| Respiratory failure  (n = 33) | (n = 22)  19= severe pneumonia  3= septic shock /acute respiratory distress syndrome | (n = 11)  11= severe pneumonia |
