# Supplementary material for: Occurrence of pendelluft during ventilator weaning with T piece correlated with increased mortality in difficult-to-wean patients
Source: J Intensive Care. 2024 Jun 24;12:23. doi: 10.1186/s40560-024-00737-z (PMC11194869; doi:10.1186/s40560-024-00737-z)
Supplement: Supplementary file 2 — Supplementary Material 2. [file 40560_2024_737_MOESM2_ESM.pdf]

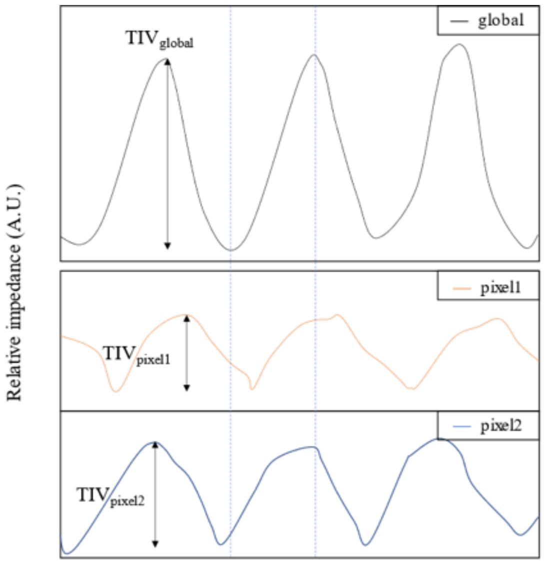

$$\text{pendelluft amplitude (\%)} = [(\sum_{i=1}^{1024} TIV_i) - TIV_{global}\ ] \,/\, TIV_{global} \times 100\%$$

Figure S1. Schematic diagram of EIT-measured pendelluft amplitude.

Pixel 1 and 2 are impedance-time curves from two representative pixels with large ventilation shift. The EIT-based pendelluft amplitude is calculated as the impedance difference between the sum of all pixel TIV and the global TIV. TIV, tidal impedance variation. A.U., arbitrary unit.
